# Efficacy and Safety of Neostigmine Adjunctive Therapy in Patients With Sepsis or Septic Shock: A Randomized Controlled Trial

**DOI:** 10.3389/fphar.2022.855764

**Published:** 2022-03-07

**Authors:** Mona M. El-Tamalawy, Moetaza M. Soliman, Amany F. Omara, Amal Rashad, Osama M. Ibrahim, Mamdouh M. El-Shishtawy

**Affiliations:** ^1^ Department of Clinical Pharmacy and Pharmacy Practice, Faculty of Pharmacy, Mansoura University, Mansoura, Egypt; ^2^ Department of Anesthesiology and Surgical Intensive Care, Faculty of Medicine, Tanta University, Tanta, Egypt; ^3^ Department of Anesthesia and Intensive Care, Faculty of Medicine, Mansoura University, Mansoura, Egypt; ^4^ Department of Clinical Pharmacy, Faculty of Pharmacy, Tanta University, Tanta, Egypt; ^5^ Department of Biochemistry, Faculty of Pharmacy, Mansoura University, Mansoura, Egypt

**Keywords:** cholinergic anti-inflammatory pathway, choline esterase inhibitor, procalcitonin, sepsis, septic shock, SOFA score

## Abstract

**Background:** Neostigmine has been found to improve survival in animal models of sepsis. However, its feasibility, efficacy, and safety in patients with sepsis or septic shock have not been investigated.

**Aim:** This parallel randomized controlled double-blinded design aimed to investigate the efficacy and safety of neostigmine as an adjunctive therapy in patients with sepsis or septic shock.

**Patients and Methods:** A total of 167 adult patients with sepsis or septic shock were assessed for eligibility; 50 patients were randomized to receive a continuous infusion of neostigmine (0.2 mg/h for 120 h; neostigmine arm) or 0.9% saline (control arm) in addition to standard therapy. The primary outcome was the change in Sequential Organ Failure Assessment (SOFA) scores 120 h after therapy initiation. Secondary outcomes included mortality rates and changes in procalcitonin level.

**Results:** The median (interquartile range) change in SOFA scores improved significantly in the neostigmine arm [−2 (−5, 1)] as compared with the control arm [1.5 (0, 2.8); *p* = 0.007]. Progression from sepsis to septic shock was more frequent in the control arm (*p* = 0.01). The incidence of shock reversal in patients with septic shock was significantly lower in the control arm than in the neostigmine arm (*p* = 0.04). Differences in 28-days mortality rates did not reach statistical significance between the control and neostigmine arms (*p* = 0.36). Percentage change in procalcitonin levels was similar in both arms (*p* = 0.74).

**Conclusion:** Neostigmine adjunctive therapy may be safe and effective when administered in patients with sepsis or septic shock.

Clinical Trial Registration: NCT04130230.

**GRAPHICAL ABSTRACT:**
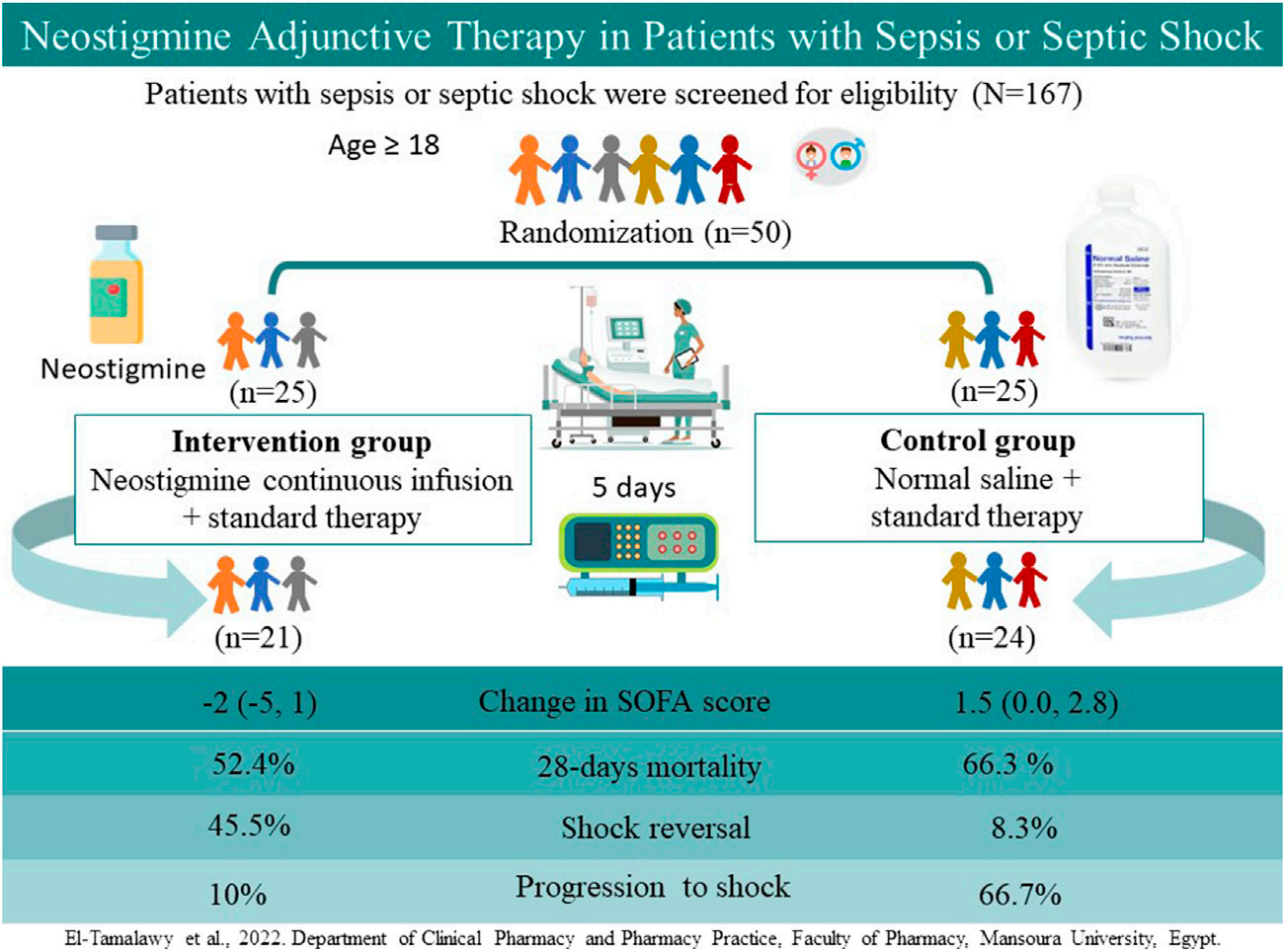


## 1 Introduction

Sepsis is a life-threatening sequential organ failure resulting from an unregulated host response to infection, whereas septic shock is a subgroup of sepsis characterized by underlying circulatory, cellular, and metabolic abnormalities that augment short-term mortality (Rhodes et al., 2016). In a recent meta-analysis including studies from North America and Europe, the incidence of septic shock–related mortality was 52.1% in hospitals (Vincent et al., 2019). Significant regional variations are observed in sepsis incidence and mortality; about 85% of cases worldwide occurred in low- and middle-income countries (Rudd et al., 2020).

Currently, the only available sepsis-specific therapy is infection control with antibiotics and drainage of the infection source if necessary. Given the high morbidity and mortality rates of septic shock, new therapeutic approaches that focus on modifying the unregulated host response to infection added to standard therapy are required to decrease mortality rates and improve patient outcomes (Hwang et al., 2020).

The cholinergic anti-inflammatory pathway (CAP) has been a focus of interest for years (Borovikova et al., 2000; Bencherif et al., 2011). CAP has been shown to be a crucial regulator of inflammation in different diseases including inflammatory bowel disease, rheumatoid arthritis, and sepsis. The concept is based on the inflammatory reflex of the vagus nerve through the suppression of systemic inflammation and protection against cytokine-mediated diseases (Wang et al., 2021). This suppression was found to be mediated by nicotinic alpha7 acetylcholine receptors (α7nAChR) on immune cells and macrophages (Wang et al., 2009).

Previous researchers have reported that in animal models of sepsis, treatment with centrally acting cholinesterase inhibitors, including physostigmine, significantly reduced mortality, offered direct stimulation of the CAP, significantly decreased the binding activity of nuclear factor–kappa B, and reduced the concentration of tumor necrosis factor–α, interleukin-1β, and interleukin-6. In addition, animals which received the peripherally acting cholinesterase inhibitor neostigmine showed similar effects as compared with animals which received physostigmine (Hofer et al., 2008). Similarly, the combined administration of neostigmine and anisodamine significantly controlled inflammation and increased survival rates in rats with induced endotoxic shock through the activation of the CAP (Sun et al., 2012).

The therapeutic effects of cholinesterase inhibitors on the functions of polymorphonuclear neutrophils (PMN) have been investigated, because PMN play a critical role in early sepsis. Physostigmine, but not neostigmine, was shown to have a direct immunomodulatory effect on PMN function (Bitzinger et al., 2013). However, in a more recent study, both physostigmine and neostigmine offered significantly protective effects on PMN functions 20 h after sepsis induction in rats and physostigmine significantly improved survival times (Bitzinger et al., 2019).

Moving from bench to bedside, the centrally acting cholinesterase inhibitor physostigmine was found to be feasible, safe, and effective in maintaining greater hemodynamic stability in patients with septic shock when administered as a continuous infusion for up to 5 days (Pinder et al., 2019). However, further studies are required to prove the efficacy of physostigmine in recovery from septic shock.

To date, no studies have investigated the role of neostigmine in human patients with sepsis or septic shock. Therefore, the current study aimed to compare the feasibility, efficacy, and safety of neostigmine in addition to standard therapy *vs.* standard therapy alone on the change of Sequential Organ Failure Assessment (SOFA) scores as a primary outcome in patients with sepsis or septic shock.

## 2 Patients and Methods

### 2.1 Trial Design

This study was a double-blind, randomized controlled trial. The study was performed in compliance with the “Declaration of Helsinki” principles and its amendments in medical research (World Medical Association, 2013). Patients were recruited from two academic teaching hospitals in Egypt: Tanta University Teaching Hospital and Mansoura University Teaching Hospital. Ethical approval was granted from the ethical committee of the Faculty of Pharmacy, Mansoura University (Code 2019-3; Clinicaltrial.gov registration No. NCT04130230). Informed consent from either the patient or legal guardian was obtained before enrollment.

### 2.2 Trial Population

Patients eligible for inclusion were: male or female adult patients, aged 18 years or older, with sepsis or septic shock after surgery according to the 2016 third international consensus definitions for sepsis and septic shock (Singer, 2016).

Patients were excluded if they had: 1) documented hypersensitivity to choline esterase inhibitors; 2) absolute contraindications to cholinesterase inhibitors such as depolarization block by depolarizing muscle relaxants, myotonic dystrophy, closed craniocerebral trauma, and mechanical obstruction in the gastrointestinal or urinary tract; 3) recognized relative contraindications to cholinesterase inhibitors such as asthmatic patients, bradycardia, and disturbance in atrioventricular conduction; or 4) previous solid organ transplantation, chronic end-stage renal failure and receiving dialysis, pregnant and lactating women, previous cardiac arrest before enrollment, presence of primary or concomitant illness approaching death, or Acute Physiologic Assessment and Chronic Health Evaluation II (APACHE II) score ≥34 (due to high predicted mortality of 80%) (Bouch et al., 2008). Patients were also excluded if they were enrolled in any another clinical trial.

### 2.3 Trial Intervention

Eligible patients were randomly assigned in an equal ratio to either the neostigmine arm or the control arm. The randomization sequence was generated by random allocation software using block randomization, stratified to sepsis and septic shock to allow for a balanced distribution of both conditions between arms.

In the neostigmine arm, neostigmine methyl sulfate (Epistegmin^®^ 0.25% vial, EPICO, Tenth of Ramadan City, Egypt), 5 mg/day was diluted in 50 ml 0.9% sodium chloride and administered as continuous infusion using an infusion syringe pump at a rate of 0.2 mg/h (2 ml/h) for 5 days without interruption. Due to lack of evidence about approved neostigmine infusion dose, the dose and duration used in the study of Pinder *et al.* was used in the current study (Pinder et al., 2019). In the control arm, patients received normal saline as placebo. All patients and outcomes’ assessors were blind to treatment allocation. The infusion syringe pump in both arms was prepared by intensive care unit (ICU) nurses who did not participate in the study. The same type of syringe pump was used for all the patients in both study arms.

### 2.4 Standard Therapy

Management other than study intervention/placebo administration were provided for all patients in accordance to surviving sepsis campaign guidelines (Rhodes et al., 2016). Intravenous broad-spectrum empiric antibiotics were administered directly after sepsis or septic shock was recognized. Blood cultures were obtained from all patients before administration of empiric antibiotics, and antibiotics were changed if needed based on sensitivity results. Surgical interventions to control the source of infection were also implemented directly when required.

### 2.5 Clinical Data Collection

#### 2.5.1 Baseline Characteristics

The baseline characteristics of the patients were collected at the time of enrollment. The collected data included age, sex, ratio of septic shock to sepsis, ratio of ventilated to nonventilated patients, source of infection, vital signs, laboratory data, arterial blood gas (ABG), fraction inspired oxygen to partial pressure of oxygen, APACHE II score (Knaus et al., 1985), Glasgow Coma Scale score (Teasdale and Jennett, 1974), and SOFA scores (Vincent et al., 1996). The level of procalcitonin (PCT) was also measured at this time (Dandona et al., 1994).

#### 2.5.2 Outcome Measures

The primary outcome was change in SOFA score (ΔSOFA), which was calculated by subtracting the final SOFA score at 120 h (5 days) from the corresponding initial value at enrollment. If the patient died within 120 h after enrollment, the worst score collected before death was used in the analysis.

Secondary outcomes included 7-days mortality, 28-days mortality, ICU length of stay, and percentage of shock reversal. Reversal of shock was defined as maintaining a stable systolic arterial pressure (>90 mm Hg) for ≥24 h without catecholamine or resuscitation fluid infusion (Bollaert et al., 1998). Shock reversal was assessed for 28 days follow up period or until patient was discharged or died. Besides, at 120 h after enrollment the following secondary outcomes were measured; percentage of progression to septic shock from sepsis, mean increase in vasopressor dose, percentage of patients who required combined vasopressors, percentage of patients with increased vasopressor dose, percentage reduction of PCT, percentage of patients with reduced PCT level, percentage of patients who achieved ≥50% reduction in PCT, and change in lactate. For lactate, if the patient died within 120 h after enrollment, the last follow-up values were used.

Safety outcomes included the percentage of patients who required atropine, percentage of patients who were reventilated, percentage of patients with hyperglycemia (blood glucose >300 mg/dl or new insulin infusion during 120 h after enrollment), hypernatremia (serum sodium >150 mmol/L during 120 h after enrollment), serious allergic reaction such as anaphylaxis, and any other unexpected adverse event.

#### 2.5.3 PCT Measurement

The PCT level was measured at baseline and at 120 h after enrollment using a standard phlebotomy procedure. The serum sample was separated by centrifugation and measured by Suresign Finecare^™^ FIA Procalcitonin Rapid Test (Suresign Professional, United Kingdom). This method is based on the fluorescence immunoassay principle and is used professionally in hospitals and laboratories. The measuring range was 0.1–100 ng/ml. Intralot precision was determined by using 10 test cartridges of the same batch to test the PCT and coefficient of variability (CV; ≤15%). In addition, interlot precision was determined by using three random and continuous batches to test PCT and CV (≤15%).

### 2.6 Sample Size Calculations

This study is the first to investigate the effect of neostigmine in patients with sepsis or septic shock. The minimum clinically important difference in SOFA score was considered to be a two-points change (Seymour et al., 2017). Therefore, a two-point difference between arms in SOFA score at the 120-h time point was anticipated. Based on the results of a previous observational study (Marik et al., 2017)^,^ we assumed a decrease of 1 (±2) point in the control arm *vs.* 3 (±2) points in the neostigmine arm over 120 h. Using G power 3.1.9.4 software and, with these estimates, α = 0.05, a *t-*test with unequal variance, and a 90% power to detect a statistically significant difference between both arms, a sample size of 23 patients was calculated in each arm. To account for a probable dropout rate of 10%, two more patients were added in each arm making a total of 25 patients.

### 2.7 Statistical Analyses

Continuous data were described as mean ± standard deviation or median with interquartile range (IQR) as appropriate, categorical data were described as counts and percentages. Groups were compared with independent *t*-test for normally distributed continuous data, whereas the Wilcoxon rank-sum test was used for ordinal data and continuous data not normally distributed. For categorical data, Fisher exact test or chi-square test were used as appropriate.

We analyzed the duration of survival and time to discharge using the Kaplan–Meier plots and compared them using the log-rank test. A *p* value of ≤0.05 was considered statistically significant. SPSS version 28 was used for statistical analysis of the data.

## 3 Results

**FIGURE 1:**
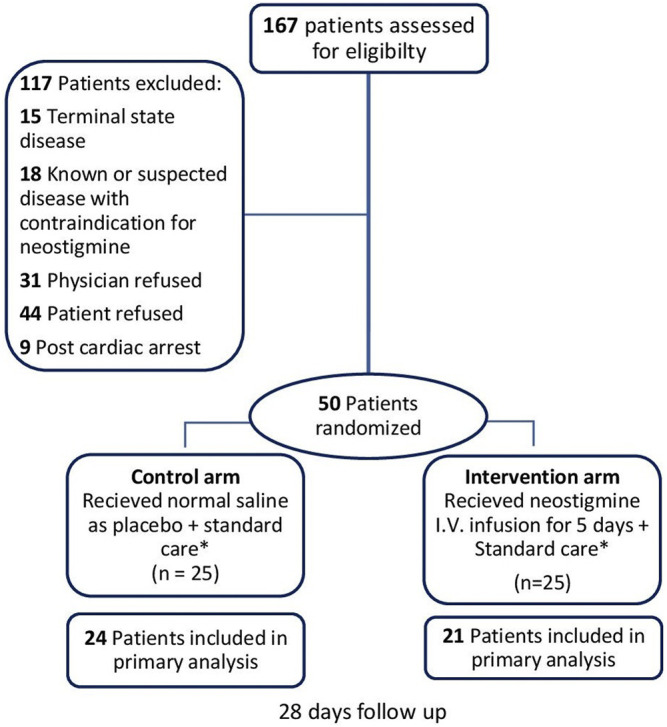
Flowchart of patient enrollment. * Management other than study intervention/placebo administration were provided for all patients in accordance to surviving sepsis campaign guidelines (Rhodes et al., 2016). Follow-up was 28 days for 28-days mortality and shock reversal, and 5 days for other outcomes.

A total of 167 patients were evaluated for eligibility; 117 patients of them were excluded (Figure 1 **)**, and the remaining 50 patients were randomized to either receive neostigmine infusion for 120 h or 0.9% saline as placebo. After 120 h, 21 patients were analyzed in the neostigmine arm *vs.* 24 patients in the control arm. In the neostigmine arm, one patient was diagnosed with COVID-19 and transferred to another ICU, one patient withdrew from study, and two patients were transferred to another ICU. In the control arm, one patient withdrew from the study.

### 3.1 Baseline Demographics and Clinical Data of the Patients

The mean age of the patients was 54.8 ± 16.3 years, and 48.9% were men. About half of the patients (51.1%) were diagnosed with septic shock, whereas 48.9% were diagnosed with sepsis. A total of 68.9% of the patients were ventilated. The most frequent site of infection was intra-abdominal (62.2%), followed by chest (20%). Other infections, including skin and central nervous system infection, accounted for 15.6%, whereas urinary tract infections accounted for 2.2%, as illustrated in Table 1. The patients’ baseline demographics and clinical data were comparable between the neostigmine and control arms (Table 1). Laboratory data, vital signs, and ABG analysis at baseline were also comparable between the two arms (Table 2). The median dose of norepinephrine was 0.1 (0, 0.2) µg/kg/min.; none of patients needed combination of vasopressors at baseline or used hydrocortisone.

**TABLE 1:** Baseline demographics and clinical data of the patients.

Variables	Control arm (*n =* 24)	Neostigmine arm (*n =* 21)	Total (*n* = 45)	*p*-value
Age, (Mean ± SD) years	52.8 ± 16.5	57.1 ± 16.2	54.8 ± 16.3	0.37 ^ϕ^
Sex, male: female (n) (%)	10:14 (41.7:58.3) %	12:9 (57.1:42.9) %	22: 23 (48.9:51.1) %	0.38^§^
Septic shock: sepsis (n) (%)	12:12 (50:50) %	11:10 (52.4:47.6) %	23: 22 (51.1:48.9) %	1^§^
Ventilated: non-ventilated (n) (%)	16:8 (66.7:33.3) %	15:6 (71.4:28.6) %	31: 14 (68.9:31.1) %	0.76^§^
Suspected infection focus:				
Intra-abdominal infection (n) (%)	16 (66.7%)	12 (57.1%)	28 (62.2%)	0.14^¥^
Chest infection (n) (%)	2 (8.3%)	7 (33.3%)	9 (20.0%)	
Urinary tract infection (n) (%)	1 (4.2%)	0 (0%)	1 (2.2%)	
Other (n) ^α^ (%)	5 (2.8%)	2 (9.5%)	7 (15.6%)	
ABG Analysis: pH, (Mean ± SD)	7.4 ± 0.08	7.4 ± 0.1	7.4 ± 0.1	0.84 ^ϕ^
PaCO_2_ (mm Hg), (Mean ± SD)	36.7 ± 9.4	36.9 ± 5.8	36.8 ± 7.9	0.9 ^ϕ^
HCO_2_ (mm Hg), (Mean ± SD)	22.5 ± 5.4	24.1 ± 5.9	23.25 ± 5.6	0.36 ^ϕ^
PaO_2_ (mm Hg), median (IQR)	73.5 (44.3, 107.3)	58.5 (33.2, 112.5)	70.8 (41.5, 107.3)	0.5 ^ϕ^
SaO_2_ (%), (Mean ± SD)	92.1 ± 8.7	89.5 ± 16.8	90.8 ± 13.2	0.57 ^ϕ^
PO_2_/FiO_2_, median (IQR)	192.9 (125.9, 214.5)	214 (82.9, 286.6)	196.4 (108.3, 278.3)	0.9 ^ϕ^
GCS ^β^, median (IQR)	10 (6.5, 14.5)	9.5 (6, 14.5)	10 (6, 15)	0.53 ^ν^
SOFA score ^δ^, median (IQR)	8.5 (5.5, 10)	8.5 (7.3, 10)	8 (7, 10)	0.44 ^ν^
APACHE II score ^µ^, median (IQR)	18 (11.5, 22.5)	19 (16, 22)	19 (12.8, 22)	0.41 ^ν^
Time from diagnosis to enrollment (hour), median (IQR)	23 (18.25, 36.25)	20 (17, 30)	22 (18, 31)	0.36 ^ν^

APACHE, acute physiology and chronic health evaluation; IQR, interquartile range; SOFA, sequential organ failure assessment; GCS, glasgow coma scale; ABG, arterial blood gas; FiO_2:_ fraction inspired oxygen.

^α^ Others refer to central nervous system infection, skin infection. ^β^ The GCS ranges from 3 (worst score) to 15 (best score); for intubated patients, it is calculated from 2 as a minimum to 10 (maximum score); for sedated patients, the last score before sedation is used. ^δ^ The SOFA score ranges from 0 to 24, with a higher score indicating higher severity of illness. ^µ^ The APACHE II score ranges from 0 to 71, with a higher score indicating higher severity of illness. ^ϕ^ Independent *t*-test was used. ^ν^ Wilcoxon rank-sum test. Pearson’s chi-square test. ^¥^ Fisher exact test.

**TABLE 2:** Laboratory data and vital signs at baseline.

Variables	Control arm (*n =* 24)	Neostigmine arm (*n =* 21)	Total (*n* = 45)	*p*-value
Vital signs				
Mean arterial pressure (mmHg), (Mean ± SD)	84.8 ± 9.6	84.9 ± 13.3	84.8 ± 11.3	0.98 ^ϕ^
Respiratory rate (per minute), (Mean ± SD)	22.4 ± 5.1	23.1 ± 5.1	22.7 ± 5	0.70 ^ϕ^
Heart rate (beat per minute), (Mean ± SD)	107.3 ± 18.6	115.5 ± 18.7	111.1 ± 18.9	0.16 ^ϕ^
Body temperature (°C), (Mean ± SD)	37.6 ± 1.1	38.2 ± 1	37.9 ± 1.1	0.09 ^ϕ^
Laboratory data at enrollment				
Hemoglobin, (Mean ± SD) (mg/dl)	9.0 ± 1.4	8.9 ± 1.5	8.9 ± 1.4	0.94 ^ϕ^
White blood cell, median (IQR) (10^3^/L)	13.3 (6.6, 19.7)	13.3 (11.8, 18.9)	13 (9.5, 18.3)	0.42 ^ν^
Platelet count, median (IQR) (10^3^/L)	254 (155, 352.5)	168 (105, 301.5)	236.5 (122.3, 329.5)	0.1 ^ν^
Total bilirubin, median (IQR), (mg/dl)	0.9 (0.5, 1.3)	0.9 (0.6, 1.6)	0.9 (0.5, 1.3)	0.55 ^ν^
Albumin, median (IQR) (g/dl)	2.5 (2.1, 3.2)	2.6 (2.3, 3)	2.5 (2.2, 3)	0.78 ^ν^
Creatinine, median (IQR) (mg/dl	0.85 (0.7, 1.8)	1 (0.8, 1.8)	0.9 (0.7, 1.7)	0.5 ^ν^
Lactate, median (IQR) (mmol/L)	2.3 (1.2, 4.2)	2.3 (1.7, 6.2)	2.25 (1.5, 3.9)	0.37 ^ν^
Random blood sugar, (Mean ± SD) (mg/dl)	150.3 ± 77.5	161.33 ± 59.7	155.38 ± 69.2	0.62 ^ϕ^
Procalcitonin (mmol/L), median (IQR)	9.94 (3.3, 22.2)	3.90 (2.2, 18.1)	7.6 (2.8, 19.9)	0.32 ^ν^
Vasopressor dose^a^ median (IQR) (µg/kg/min.)	0.1 (0, 0.2)	0.15 (0, 0.3)	0.1(0, 0.2)	0.36 ^ν^

IQR, interquartile range; ABG, arterial blood gas. ^ϕ^ Independent *t*-test. ^ν^ Wilcoxon rank-sum test.

a Noradrenaline is used as vasopressor.

### 3.2 Primary Outcome

**FIGURE 2:**
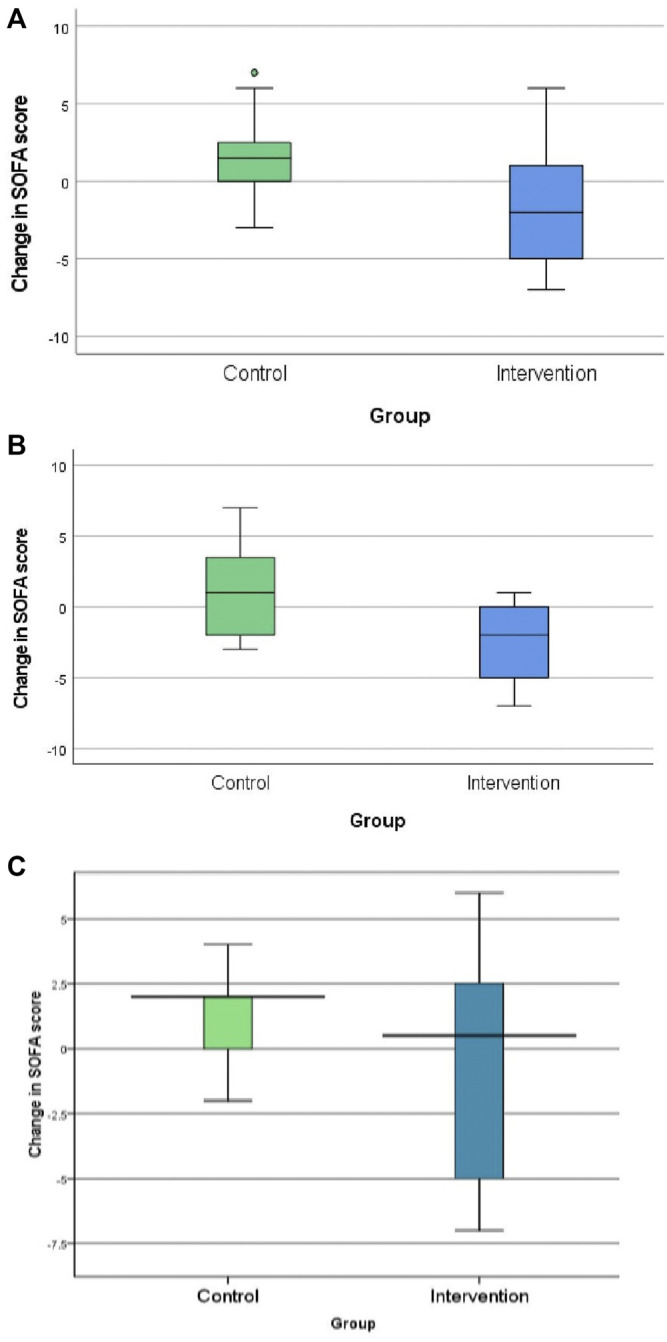
Change in Sequential Organ Function Assessment (SOFA) score in the control and neostigmine arms. **(A)** In all patients. **(B)** In patients with sepsis. **(C)** In patients with septic shock. *p-value <0.05.

**FIGURE 3:**
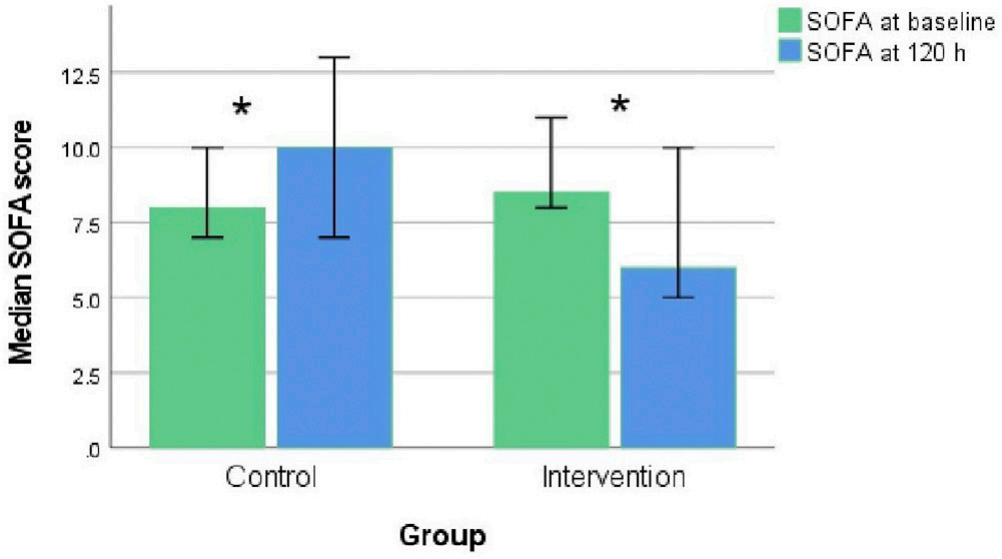
SOFA scores at baseline and at 120 h in the control and neostigmine arms. *p < 0.05.

The neostigmine arm showed significant improvements in SOFA score change at 120 h [−2.0 (IQR −5, 1)], while the control arm showed no improvement [1.5 (IQR 0.0, 2.8); *p* = 0.007], as shown in Figure 2A; Table 3. Figure 3 shows the SOFA scores at baseline compared with 120 h after enrollment in the neostigmine and control arms. Looking at the SOFA subscores, there was a statistically significant improvement in cardiovascular function with neostigmine [0 (IQR 0.0, 4.0)] as compared with the control arm [4 (IQR 1.5, 4.0), *p* = 0.03]. However, as illustrated in Table 4, other subscores did not show significant differences. In the subgroup analysis including only patients with sepsis, the change in SOFA score at 120 h showed a statistically significant improvement in the neostigmine arm in comparison with the control arm (*p* = 0.02), as shown in Figure 2B. On the other hand, this improvement did not reach statistical significance in patients with septic shock (*p* = 0.5; Figure 2C).

**TABLE 3:** Primary and secondary outcomes.

Outcome	Control arm (*n =* 24)	Neostigmine arm (*n =* 21)	*p*-value
Final (120 h) SOFA score, median (IQR)	10 (5.5, 13)	6 (4.8, 10.3)	0.07 ^ν^
ΔSOFA score, median (IQR)	1.5 (0, 2.8)	−2 (−5, 1)	0.007 ^ν^
7-days mortality, No. /total (%)	8/24 (33.3%)	5/21 (23.8%)	0.53 ^¥^
28-days mortality, No. /total (%)	16/24 (66.7%)	11/21 (52.4%)	0.37 ^¥^
Shock reversal, No. /total (%)	1/12 (8.3%)	5/11 (45.5%)	0.04^§^
Progression to septic shock, No. /total (%)	8/12 (66.7%)	1/10 (10%)	0.01 ^¥^
Need for vasopressor ^α^, No. /total (%)	18/24 (75%)	9/21 (42.9%)	0.04 ^¥^
Patients with increased vasopressor dose, No. /total (%)	17/24 (70.8%)	8/21 (38.1%)	0.04 ^¥^
Dose of vasopressor, median (IQR) (µg/kg/min.) ^β^	0.2 (0, 0.6)	0 (0, 1)	0.29 ^ν^
Need for combined vasopressors, No. /total (%)	3/24 (12.5%)	0/21 (0%)	0.2 ^¥^
Procalcitonin (PCT)			
PCT at 120 h (mmol/L), median (IQR)	8.3 (2.8, 24.7)	4.2 (0.71, 16.1)	0.24 ^ν^
Δ PCT, median (IQR) ng/ml	0 (−2, 0)	−1.39 (−2.9, 0.9)	0.26 ^ν^
% Reduction, median (IQR)	0 (−68.5%, 0%)	−50% (−76%, 0%)	0.19 ^ν^
Patients who achieved > 50% reduction, No. /total (%)	6/12 (50%)	8/13 (61.5%)	0.7 ^¥^
Patients with increased PCT, No. /total (%)	5/12 (41.7%)	3/13 (23.1%)	0.41^¥^
120 h Lactate, median (IQR) (mmol/L)	1.8 (1.3, 5.6)	1.8 (0.9, 2.14)	0.5^ν^
Change in Lactate, median (IQR) (mmol/L)	0.8 (0.1, 2.2)	0 (−1.2, 0.9)	0.07 ^ν^
Creatinine, median (IQR) mg/dl (*n* = 33)	1.2 (0.9, 2.3)	0.7 (0.5, 0.9)	0.002 ^ν^
Length of ICU stay, median (IQR) (days)	18 (7, 25)	7 (6, 10)	0.21 ^ν^
Patients discharged, No. /total (%)	8/24 (33.3 %)	10/21 (47.6 %)	0.32^§^

ΔSOFA, change in sequential organ failure assessment score; PCT, procalcitonin.

^ν^ Wilcoxon rank-sum test. ^¥^ Fisher exact test. ^§^ Pearson’s chi-squared test. ^ϕ^ Independent *t*-test. ^α^ Patients who stopped vasopressor after 120 h of enrollment are counted zero. ^β^ Noradrenaline is used as vasopressor.

**TABLE 4:** Subscores of Sequential Organ Function Assessment (SOFA) score at enrollment and after 120 h.

SOFA sub-score	Control arm		Neostigmine arm		*p-value*
	At baseline	At 120 h	At baseline	At 120 h	
Respiratory	3 (2, 3)	3 (2, 3.75)	3 (2, 4)	2.5 (1.75, 3)	0.81*
					0.20**
Coagulation	0 (0, 0)	0 (0, 1)	0 (0, 1)	0 (0, 1.25)	0.15*
					1**
Hepatic	0 (0 ,0)	0 (0, 1)	0 (0, 0)	0 (0, 0)	0.93*
					0.6**
Cardiovascular	3 (0, 4)	4 (1.5, 4)	3 (0, 4)	0 (0, 4)	0.47*
					0.03**
Central nervous system	2 (0.25, 3)	2.5 (1.25, 3)	3 (0.75, 3)	3 (0, 3)	0.31*
					0.93**
Renal	0 (0, 1)	0 (0, 1.75)	0 (0, 1)	0 (0, 0)	0.93*
					0.23**

Data are expressed as median (interquartile range). Wilcoxon rank-sum test is used.

*p value for comparing subscore values at baseline. **p value for comparing subscore values at 120 h.

### 3.3 Secondary Outcomes

#### 3.3.1 Mortality

The neostigmine arm had lower 7-days mortality and 28-days mortality rates than the control arm did, although the difference was not statistically significant (23.8 *vs.* 33.3%, *p* = 0.53, and 52.4 *vs.* 66.3%, *p* = 0.37, respectively; Table 3).

**FIGURE 4:**
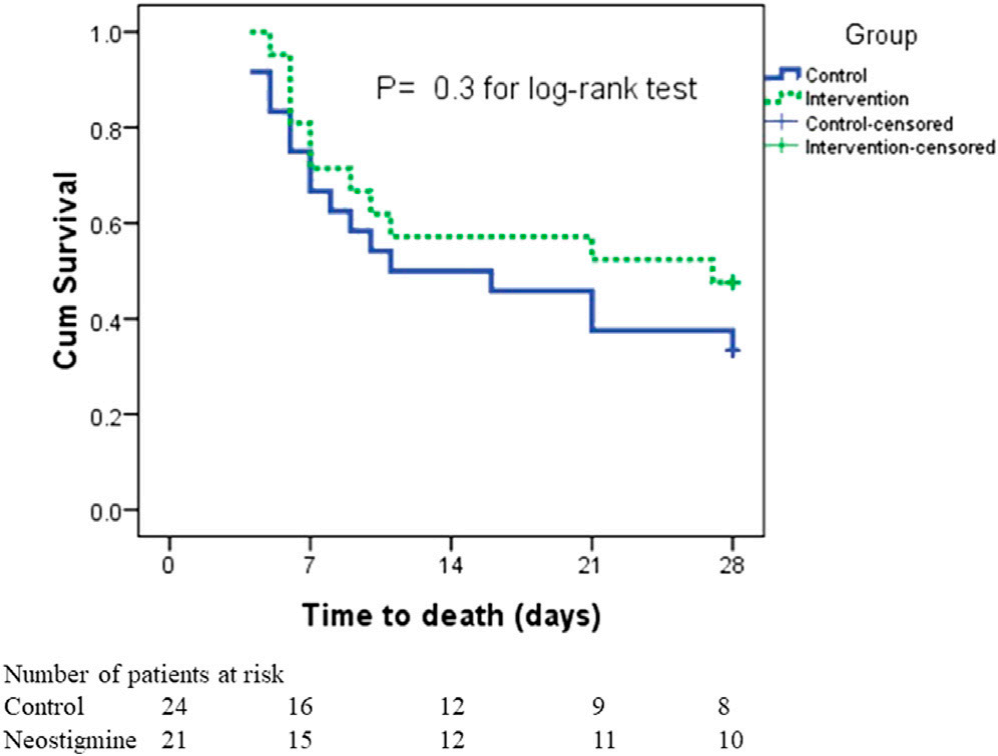
Kaplan–Meier survival estimates of 28-days mortality.

In the survival analysis, taking into consideration both the incidence of death and time to death, there was no statistically significant difference in survival rates between the two arms; the mean time to death was 18.8 ± 2.2 days in the neostigmine arm compared with 16.3 ± 2.1 days in the control arm (Figure 4). By the seventh day, six patients had died in the neostigmine arm, whereas eight patients died in the control arm. In addition, by day 21, an additional four deaths were reported in the neostigmine arm, whereas seven additional deaths were reported in the control arm. By day 28, only one more patient had died in the neostigmine arm, to yield 11 deaths overall (28-days mortality) in the neostigmine arm. On the other hand, an additional two patients died in the control arm, resulting in 16 deaths overall (28-days mortality) in the control arm (*p* value for log-rank test = 0.3).

#### 3.3.2 Length of ICU Stay

**FIGURE 5:**
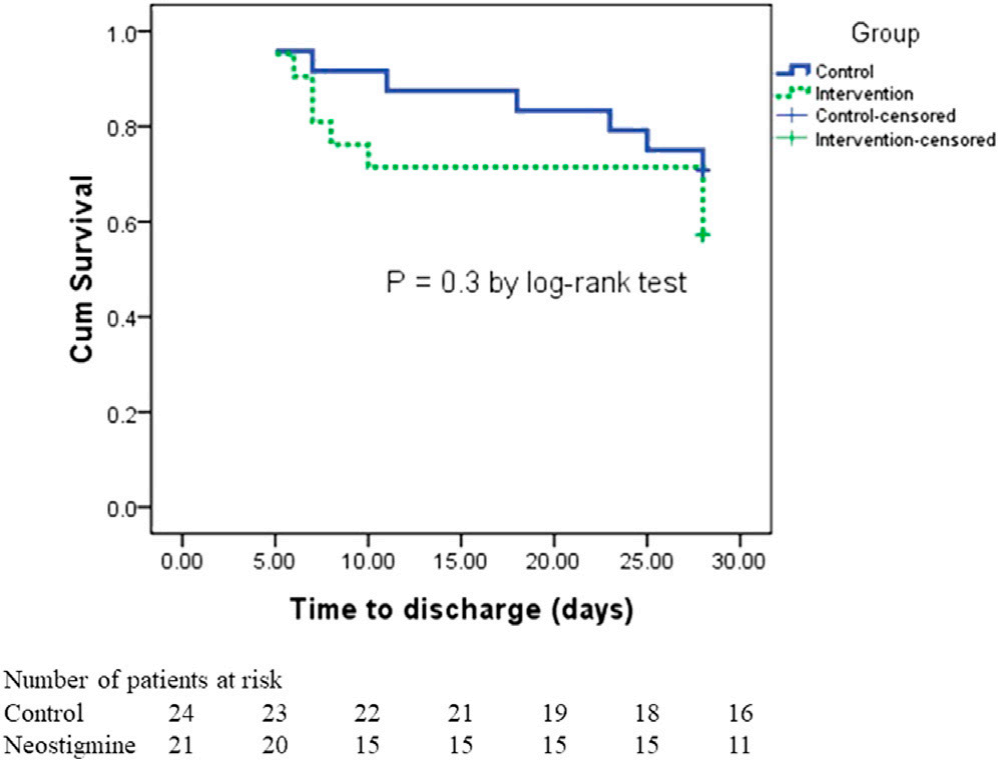
Kaplan–Meier survival estimates of ICU discharge.

A total of 47.6% of patients in the neostigmine arm were discharged compared with 33% of patients in the control arm (*p* = 0.3). The length of hospital stay did not differ between the neostigmine arm and the control arm (*p* = 0.21). The median (IQR) days to discharge was 7 (6, 10) days in the neostigmine arm compared with 18 (7, 25) days in the control arm, (*p* = 0.2; Table 3). Figure 5 shows the survival analysis for time to discharge during the 28-days follow-up period. The log-rank test showed no differences between the neostigmine and control arms (*p* = 0.3).

#### 3.3.3 Septic Shock

In patients with septic shock, there was a statistically significant difference in shock reversal between the neostigmine and control arms (45.5 and 8.3% respectively, *p* = 0.04). In addition, progression from sepsis to septic shock was significantly lower in the neostigmine arm compared with the control arm (10 and 66.7%, respectively; *p* = 0.01; Table 3).

Serum lactate level was decreased in the neostigmine arm compared with the control arm [0 (−1.1, 0.5) and 0.8 (1.0, 2.2), respectively, *p* = 0.09]. However, creatinine level at 120 h was significantly lower in the neostigmine arm than in the control arm [0.7 (0.5, 0.9) *vs.* 1.2 (0.9, 1.8), respectively*, p* = 0.004].

**FIGURE 6:**
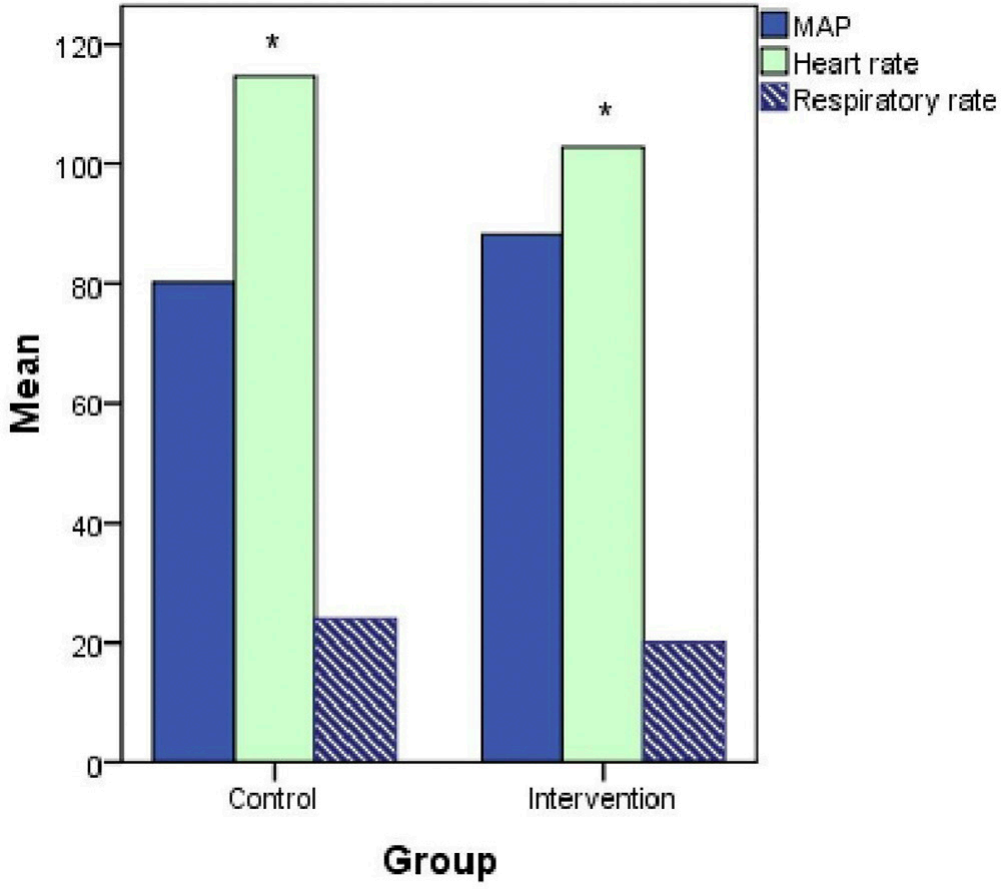
Hemodynamics (mean arterial pressure [MAP], heart rate, and respiratory rate) at 120 h after treatment initiation in patients with septic shock.

In the subgroup analysis, neostigmine improved the hemodynamics in patients with septic shock: mean arterial pressure (MAP), 88.3 ± 9.1 *vs.* 80.2 ± 12.6 (*p* = 0.17); heart rate, 97 ± 16 *vs.* 115 ± 16 (*p* = 0.05); and respiratory rate, 20 ± 4 *vs.* 24 ± 7 (*p* = 0.2; Figure 6).

#### 3.3.4 Use of Vasopressor

Neostigmine significantly decreased the percentage of patients who still required vasopressors at 120 h after enrollment (42.9% in the neostigmine arm compared with 75% in the control arm, *p* = 0.04). Similarly, neostigmine decreased the percentage of patients requiring a higher vasopressor dose at 120 h (38.1% in the neostigmine arm compared with 70.8% in the control arm, *p* = 0.04). However, there was no significant difference in the mean dose of vasopressor between the neostigmine and control arms, although the dose was higher in the control arm (0.29 ± 0.44 and 0.37 ± 0.36, respectively, *p* = 0.52). In addition, no patients required combined vasopressor in the neostigmine arm, whereas 12% of patients in the control arm did (*p* = 0.2; Table 3).

#### 3.3.5 PCT Level

The percentage decrease in PCT was higher in the neostigmine arm than in the control arm, but this difference was not statistically significant [−50% (−76%, 0%) and 0% (−68.5%, 0%), respectively, *p* = 0.74]. In addition, the percentage of patients who achieved a ≥50% reduction in PCT was 61.5 *vs.* 50%, respectively (*p =* 0.7). On the other hand, the percentage of patients with increased PCT was lower in the neostigmine arm compared with the control arm (23.1 *vs.* 41.7%, respectively, *p* = 0.4; Table 3).

### 3.4 Safety

Considering adverse effects, the administration of a continuous infusion of neostigmine for 120 h was well tolerated, and no early termination or decrease in the rate of infusion was required, except for one patient who developed bradycardia 2 h after the start of the infusion. The effect on heart rate was comparable with the control arm, with no statistically significant difference between the intervention and control arm (105 ± 18 *vs.* 103 ± 13, respectively, *p* = 0.6). Other adverse events were minor: one patient developed fits once during the treatment period with no medication needed, and one patient developed hypertension, which was resolved with the angiotensin converting enzyme inhibitor captopril (Capoten^®^ 25 mg) for 1 day only.

No patient in either arm was reventilated or required atropine to reverse the cholinergic effect of neostigmine.

## 4 Discussion

In the current study, we investigated the efficacy and safety of neostigmine as an adjunctive therapy in patients with sepsis or septic shock.

A continuous infusion of neostigmine for 120 h significantly improved SOFA scores in the neostigmine arm as compared with the control arm, indicating that neostigmine helped to improve sequential organ dysfunction associated with sepsis and septic shock. In the study by Pinder et al. of physostigmine, the mean SOFA score was 8.9 ± 2.5 for the physostigmine arm and 11.1 ± 3.8 for the placebo arm (*p* = 0.19), using age as a covariate (Pinder et al., 2019). However, the results of the physostigmine study were statistically insignificant, which might be because of the smaller sample size (N = 20). In addition, that study was restricted to patients with septic shock, who have a worse prognosis than sepsis patients do. However, those findings still support our results indicating that cholinesterase inhibitors may improve organ dysfunction in patients with sepsis and septic shock. In our study, the improvement in organ dysfunction was also reflected by a statistically significant difference in shock reversal between the neostigmine arm and control arm. In the subgroup analysis, neostigmine improved the SOFA score in patients with sepsis. On the contrary, when the analysis was limited to patients with septic shock, this improvement did not reach statistical significance.

In the current study, the administration of neostigmine was associated with a statistically significant improvement in decreasing the progression from sepsis to septic shock as compared with control arm, as only 10% of the patients progressed to shock in the neostigmine arm vs 66.7% in the control arm. Acetylcholine and nAChR agonists inhibit the synthesis of proinflammatory cytokines on immune cells and hence protect against septic shock (Tracey, 2010). These results, when added to the subgroup analysis of the SOFA score mentioned above, can be explained by the inhibitory effect of neostigmine on proinflammatory cytokines in early sepsis. Consequently, the early use of neostigmine may show better efficacy compared with late administration.

The significant improvement in cardiovascular function subscore in the neostigmine arm indicates that the efficacy of neostigmine in sepsis and septic shock is mainly related to its effect on hemodynamics. A significantly lower percentage of patients taking neostigmine required vasopressor after 120 h, and consequently, a lower percentage of patients required an increased vasopressor dose compared with the control arm. No patient required combined vasopressor in the neostigmine arm, whereas 12.5% of patients in the control arm did; however, this difference was not statistically significant. Although the mean dose of vasopressor was lower in the neostigmine arm, it was still statistically insignificant. This result can be explained by the relatively small sample size; in addition, the doses of vasopressors were very small. Hence, a very large sample size is required to detect a statistically significant difference. Consequently, the percentage of patients requiring vasopressor 120 h after enrollment, as mentioned above, is a better reflection. Similar results regarding hemodynamics have been reported in a physostigmine study; the mean dose of vasopressor (norepinephrine) in the physostigmine arm was slightly lower than in the control arm [0.2 (0.1) *vs.* 0.3 (0.1), respectively, *p* = 0.06] (Pinder et al., 2019). In addition, the percentage of patients who required combined vasopressors was 20% in the physostigmine arm *vs.* 50% in the control arm, but only dobutamine showed a significant difference.

Although not statistically significant, at baseline the median PCT in the control arm was more than double that in the neostigmine arm. This may be attributed to the respiratory focus of infection in neostigmine arm and the urogenital focus of infection in the control arm (Thomas-Rüddel et al., 2018). The results of the current trial showed no statistically significant difference in procalcitonin level 120 h after enrollment. This can be attributed to the small number of patients with a measured PCT after 120 h (12 of 24 patients in the control arm *vs.* 13 of 21 patients in the neostigmine arm). Similarly, Pinder et al. did not show a statistically significant difference between the control arm and physostigmine arm. For most patients in both arms, PCT dropped to below 1 ng/ml at the eighth visit (Pinder et al., 2019).

Serum creatinine was significantly improved in the neostigmine arm compared with the control arm. However, this improvement in kidney function was not reflected in the renal subscore of SOFA. Using scoring system may underestimated the difference in creatinine between neostigmine and control arms (Altman and Royston, 2006). Experimental evidence demonstrating that vagal nerve stimulation can reduce renal inflammation and protect the kidney from acute ischemic injury (Jarczyk et al., 2019; Kamel et al., 2021). Accordingly, in patients with sepsis, neostigmine may have a prophylactic effect on kidney dysfunction.

Physicians concerns about the possible cardiovascular and respiratory adverse effects of neostigmine (Neostigmine Adverse Effects, 2022) may have limited the number of patients recruited in this study. Considering the adverse effects of neostigmine, one patient developed bradycardia at 2 h after starting the infusion. Although neostigmine can cause bradycardia, the cause of neostigmine could not be confirmed in this patient, as no rechallenge test was conducted. In addition, the patient was taking the antidepressant amitriptyline and the antifungal fluconazole, which have been reported to cause irregular heartbeats. Moreover, the patient’s laboratory data showed hypokalemia, which is another contributor to irregular heartbeats and a risk factor for Torsades de pointes, which implies heart rhythm disturbances that can be life-threatening (Ward et al., 1975). The second serious adverse effect was hypertension in one case, but this resolved after administration of antihypertensive for 1 day. This effect was also reported with physostigmine (Pinder et al., 2019).

To the best of our knowledge, the current trial is the first to evaluate the efficacy and safety of neostigmine as an adjunctive therapy in patients with sepsis and septic shock. Possible limitations of this study include the relatively small sample size, which may cause statistical overestimation of affect size, and early death of some patients before the course of treatment was completed. However, in those cases, the worst SOFA score collected before death was used in the analysis. Patients with other comorbidities leading to impending death were excluded, which decreased the feasibility of a patient’s recruitment. Moreover, we used a relatively low dose of neostigmine because of safety concerns of using neostigmine for the first time as a long-time continuous infusion in this population, and this added to the lack of evidence about the infusion dose for neostigmine in patients with sepsis or septic shock. Accordingly, further studies with higher doses of neostigmine infusion are recommended, if tolerated. We did not address the correlation between neostigmine and the affecting organism, because this is the first study of neostigmine in patients with sepsis and septic shock; however, additional studies are recommended to address its efficacy in different sources of infections.

## 5 Conclusion

The administration of neostigmine showed an improvement in SOFA scores in patients with sepsis and septic shock. It also helped to promote recovery from septic shock, and if taken early, it can decrease the progression from sepsis to septic shock. In patients with sepsis and septic shock, neostigmine might be a promising, low-cost adjunctive therapy. A large multi-center randomized trial is recommended to confirm these findings.

## Data Availability

The original contributions presented in the study are included in the article/Supplementary Material, further inquiries can be directed to the corresponding author.
